# Matrix-Assisted Laser Desorption/Ionization Time of Flight Mass-Spectrometry (MALDI-TOF MS) Based Typing of Extended-Spectrum β-Lactamase Producing *E*. *coli* – A Novel Tool for Real-Time Outbreak Investigation

**DOI:** 10.1371/journal.pone.0120624

**Published:** 2015-04-10

**Authors:** Adrian Egli, Sarah Tschudin-Sutter, Michael Oberle, Daniel Goldenberger, Reno Frei, Andreas F. Widmer

**Affiliations:** 1 Division of Clinical Microbiology, University Hospital Basel, Basel, Switzerland; 2 Infection Biology Lab, Department Biomedicine, University of Basel, Basel, Switzerland; 3 Division of Infectious Diseases and Hospital Epidemiology, University Hospital Basel, Basel, Switzerland; 4 Clinical Microbiology, Cantonal Hospital Aarau, Aarau, Switzerland; University of Leuven, BELGIUM

## Abstract

Epidemiologically linked clusters are confirmed by typing strains with molecular typing such as pulsed-field gel electrophoresis (PFGE). We compared six extended-spectrum β-lactamase producing *E*. *coli* of a PFGE-related cluster with Matrix-assisted laser desorption/ionization-time of flight mass-spectrometry based typing that confirmed relatedness faster and more cost-effective, but as reliable as PFGE.

## Introduction

Rapid and specific identification of strain-identity are integral components of outbreak investigation and subsequent implementation of effective infection control measures [1, 2]. Conventional molecular typing methods such as pulsed-field gel electrophoresis (PFGE) or MLST-typing are labor-intensive and require up to one week from sample collection to delivery of typing results [3–5]. Matrix-assisted laser desorption/ionization time of flight mass-spectrometry (MALDI-TOF MS) is able to identify bacterial species via determination of highly specific protein mass-spectra profiles within minutes and has revolutionized the identification of bacteria [6]. MALDI-TOF MS has a high resolution within the mass-range of 2 to 20kDa, covering predominantly ribosomal proteins demonstrating important differences at a species level [7–9]. These features may be used to rapidly assess strain relatedness. We hypothesize that (i) variance at a species level may be rapidly determined by using MALDI-TOF MS and that (ii) results reveal similar clustering when compared to conventional PFGE based typing. Therefore, we aimed to assess the validity of MALDI-TOF MS based typing in a pilot study in a previously published nosocomial outbreak of extended-spectrum β-lactamase (ESBL)-producing *Escherichia coli* with PFGE to confirm relatedness of strains [10].

## Material and Methods

We re-analysed six isolates of ESBL-producing *E*. *coli* collected during an outbreak at the University hospital Basel, Switzerland, [10] by performing MALDI-TOF MS based typing and comparing the results to those obtained by pulsed-field gel electrophoresis. Two non-related independent control ESBL *E*. *coli* strains were included.

### Pulsed-field gel electrophoresis (PFGE) typing

The PFGE method has been performed as previously published [11]. Briefly, DNA restriction fragments were separated by PFGE after *Xba* I digestion and dendrograms were drawn with use of the software GelCompar, version 4.5 (Applied Maths, Belgium).

### MALDI-TOF MS based typing

A detailed standard operating procedure is provided in **S1 Text**. Briefly, all bacterial isolates were stored at −80°C. These were thawed and sub-cultivated for re-analysis at standard conditions on a blood agar plate in an aerobic atmosphere at 37°C for 18h. All isolates were of same age to control for senescence-associated changes in the mass peak spectrum. Full protein extraction using ethanol washes and formic acid (70%) was used to increase the spectrum quality and was repeated three times independently to assess reproducibility. For each isolate, four separate spectra were recorded using the Flex Control software (Bruker Daltonics, Bremen, Germany). The measurements were performed on Microflex MALDI-TOF (Bruker Daltonics). Voltage settings were: digitizer 1000V, detector gain voltage offset linear at 2650V, and reflector at 1400V. Spectrometer ion source 1 was set at 19.98kV, source 2 at 18.08kV, and lens at 6kV. Spectra were recorded within the range of 2 to 20 kDa. Species were confirmed in comparison with the mass-spectrum library using the MALDI Biotyper 3 software (OC 3.1, Bruker Daltonics) at standard conditions. Time for performance of MALDI-TOF MS based typing was recorded.

### Bioinformatic analysis

Recorded profiles were analysed first to confirm the bacterial species using the library database including mass spectrometry profiles of 4623 pathogens. The profiles were then smoothed and baseline peak shifts were subtracted using the Biotyper 3 software. Principal component analysis (PCA) was used to determine clusters with similar protein expression by applying euclidean distance measures and single linkage algorithms. Lower bound was set at 3000 arbitrary units, upper bound at 15000 arbitrary units, and resolution was 2.

Flex Analysis software (Bruker Daltonics) was employed to identify single peaks of each isolate (see **S1 Table**). In addition, in an overlay of all isolates ‘peak shifts’ were identified between outbreak and non-related clusters. A significant peak has to be above 1000 arbitrary units and a signal-to-noise ratio of >10. Peaks between different isolates had to be separated by at least 5 m/z, but less than 50m/z, as this can reflect an unrelated peak.

## Results

### Species identification

All strains previously phenotypically identified by VITEK 2 were re-confirmed by MALDI-TOF MS (**S1A Fig**). Highly reproducible peaks were identified for each isolate by repetition of full protein extraction in three independent experiments.

### High resolution clustering of outbreak and non-related isolates with MALDI-TOF MS in comparison to PFGE based typing

**Fig 1:**
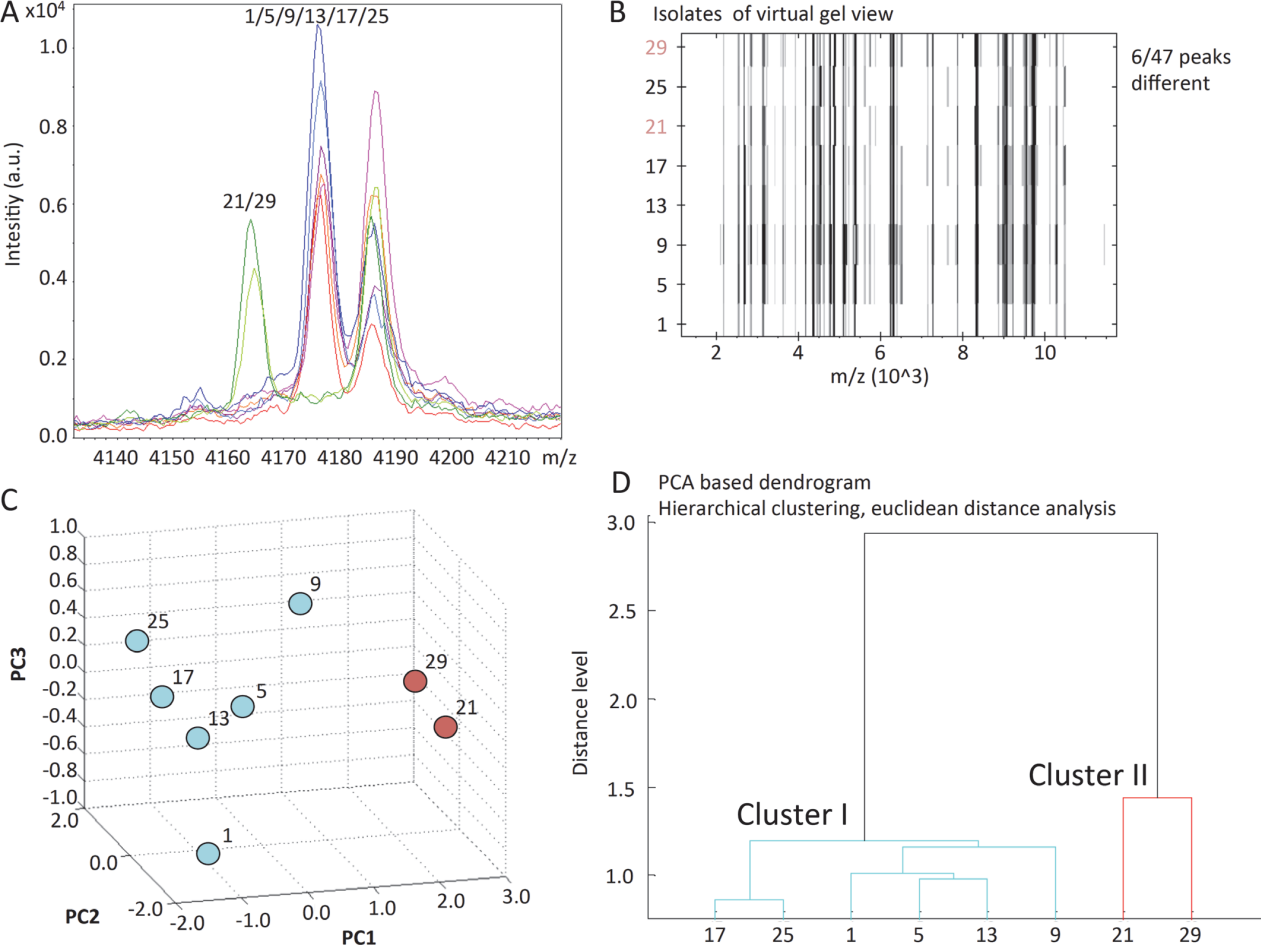
MALDI-TOF MS based typing. **A. Representative example of mass spectrum differences between ESBL *E*. *coli*.** Isolates are indicated by color code. Isolates 1, 5, 9, 13, 17 and 25 (outbreak cluster) show a clear distinguished peak at position 4175 m/z, whereas isolates 21 and 29 show a shift to position 4165 m/z (non-related cluster). This corresponds to change of about 10Da. Various other areas of peak shifts have been identified between outbreak and non-related ESBL *E*. *coli* clusters. **B. MALDI-TOF MS based virtual gel view.** MS generated peaks of the protein expression profile are shown for every bacterial isolate. Red labels highlight the non-related isolates. **C. Three-dimensional principal component analysis.** Based on the Euclidean distance analysis algorithm a three dimensional plot was generated. PC1 and PC2 showed the highest discriminatory potential and indicated a cluster for the outbreak associated isolates, whereas the non-related isolates are less similar. **D. PCA-based dendrogram generated by MALDI-TOF MS.** The PCA based dendrogram uses a hierarchical clustering algorithm with a Euclidean distance analysis.

All spectra were magnified and mass peak profiles were screened to determine typical peaks of the *E*. *coli* isolates. In total 47 peaks were identified. We then screened for differences corresponding to changes within the mass-spectrum of an individual isolate (corresponding to changes in the amino acid sequence). Typical shifts in the mass peak spectrum observed in ESBL-producing *E*. *coli* isolates are illustrated in **Fig 1A** and **S1B Fig**. Mass peaks, which were significantly shifted (>10 and <100m/z) allowing the separation of outbreak and non-related clusters are summarized in **S1 Table.** A virtual gel-view of all isolates is represented in **Fig 1B**. Principal component analysis revealed PC1 as the strongest denominator explaining the clustering with 70% (**Fig 1C**), while PC2 and PC3 explained 20% and 10% of the cluster distribution, respectively. S2A-C Fig summarize the results of a two-dimensional cluster analysis. Plotting PC1 and PC2 showed a high resolution to discriminate the outbreak and non-related clusters. Similarly a PCA-based dendrogram accurately delineates clusters (**Fig 1D**), identifying six highly related (outbreak), and two unrelated ESBL *E*. *coli* isolates. All results from MALDI-TOF MS based typing including the detailed analysis of peak frame shifts were obtained within less than one day.

**Fig 2:**
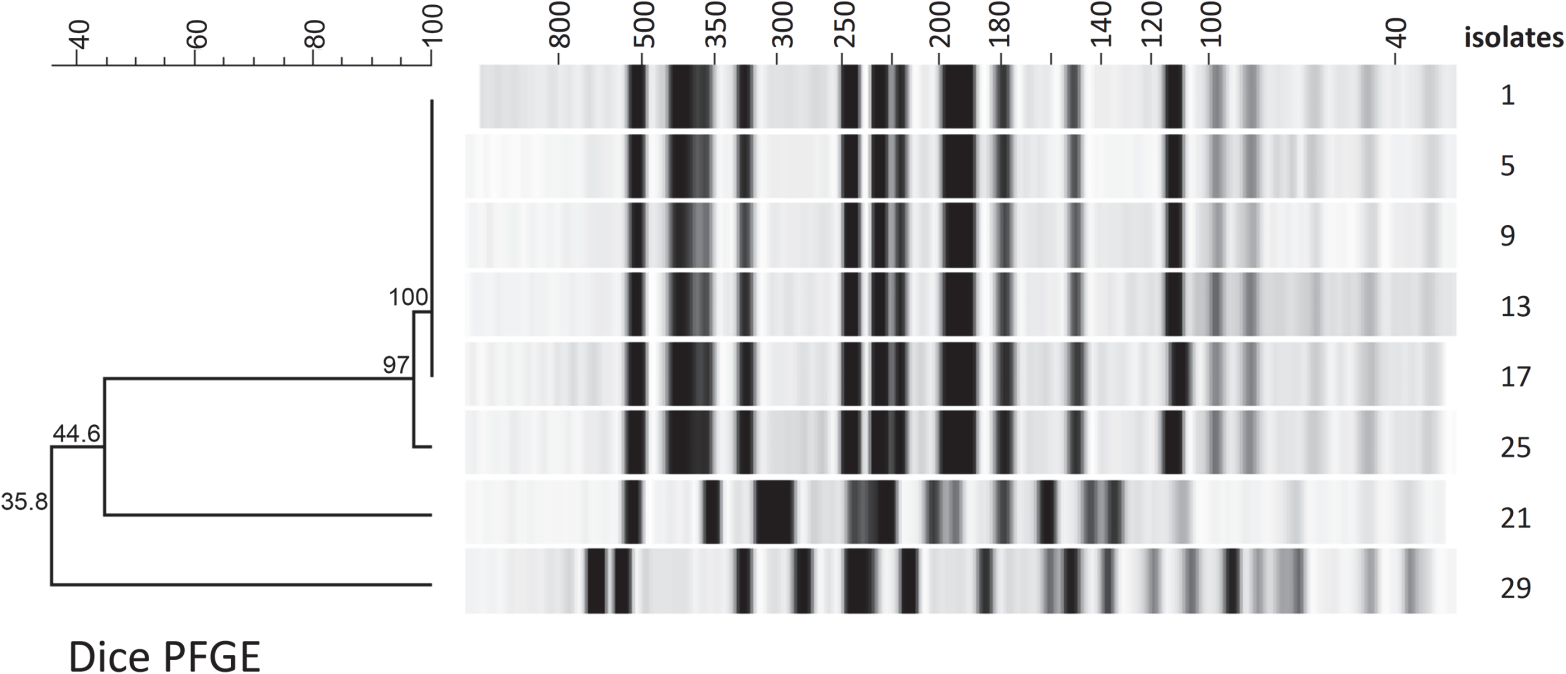
PFGE based typing with gel-view and dendrogram. Dice analysis indicates a very high relatedness in the outbreak-associated cluster. Isolates 21 and 29 are clearly separated.

The PFGE and MALDI-TOF MS based dendrograms in direct comparison are highly similar (**Fig 2**). In spite of divergent methodology—dice correlation analysis and PCA—each analytical method reliably identified the non-related cluster.

## Discussion

This MALDI-TOF MS based typing approach can reliably identify clusters of ESBL *E*. *coli* isolates arising during a hospital outbreak in a timely manner. In our study, we could obtain all results including a detailed frame shift analysis of MS peaks in less than one day compared to one week with PFGE. Typing results (in particular the PCA-based dendrogram) become available at the same time as susceptibility results, at a speed that allows adequate and efficient implementation of infection control measures.

Key steps for successful MALDI-TOF MS based typing are the same age of bacterial subcultures, a full protein extraction protocol, and inclusion of non-related isolates. Subculture of the isolates requires overnight incubation, however the full protein extraction and data analysis using the algorithms presented can be accomplished within three hours. All methods are provided in full detail in the supplementary material.

For typing, isolates representing the outbreak strain would ideally be indistinguishable from each other and highly diverse from those of non-related strains. For PFGE-based typing, significant differences have been defined as distinctions in at least seven bands [12], corresponding to an approximately 80% similarity between isolates. For MALDI-TOF based typing no similarity rate can be calculated at present and this requires future study with larger cohorts. Although for *Enterococcus faecium* and *Staphylococcus aureus* only a low discriminatory power has been reported [13], our non-related *E*. *coli* isolates were clearly separated with highly diverse peaks.

Our study has a number of limitations. First, this was a small outbreak focusing on one pathogen, which may be more conducive to taking a MALDI-TOF based approach. However, even with more samples the processing time is significantly shorter in comparison to PFGE. An important second limitation is that, the small sample size may limit generalizability of our results to other settings. However, we would like to point out that the results were highly reproducible in three independent experiments. Nevertheless, further studies comparing PFGE and MALDI-TOF on larger sample sizes are needed [14]. To the best of our knowledge, no software is currently available, which would allow the direct comparison between MALDI-TOF (protein peak data) and PFGE (DNA restriction enzyme pattern) typing data.

Overall, our pilot study highlights an impressive time gain in identifying similarities and differences between ESBL *E*. *coli* strains. This novel approach to outbreak investigation may allow real-time typing and revolutionize outbreak investigations if further larger studies are able to confirm our results. Definitions on what constitutes a significant peak change in MALDI-TOF MS for typing should be approached.

## Supporting Information

S1 Fig A. Identification of *E. coli* compared to reference library spectra. Blue lines show MS peaks of an individual isolate. Red lines indicate the reference peaks. **B. Representative example of mass spectrum differences between ESBL *E*. *coli*.** Isolates are indicated by color code. Isolates 1, 5, 9, 13, 17 and 25 (outbreak cluster) show a clear distinguished peak at position 4859 m/z, whereas isolates 21 and 29 show a shift to position 4872 m/z (non-related cluster). This corresponds to change of about 13Da.
(TIF)

S2 Fig (A-C) Two-dimensional principal component analysis (PCA) of the protein expression profiles. The influence on the data distribution by PC1, PC2 and PC3 is 70%, 20%, and 10% respectively.
(TIF)

S1 Text A detailed step-by-step protocol is provided for performing MALDI-TOF based typing. (DOCX)

S1 Table List of all identified peaks. (DOCX)
