# Supplementary figures and images for: Matrix-Assisted Laser Desorption/Ionization Time of Flight Mass-Spectrometry (MALDI-TOF MS) Based Typing of Extended-Spectrum β-Lactamase Producing E. coli – A Novel Tool for Real-Time Outbreak Investigation
Source: PLoS One. 2015 Apr 10;10(4):e0120624. doi: 10.1371/journal.pone.0120624 (PMC4393243; doi:10.1371/journal.pone.0120624)

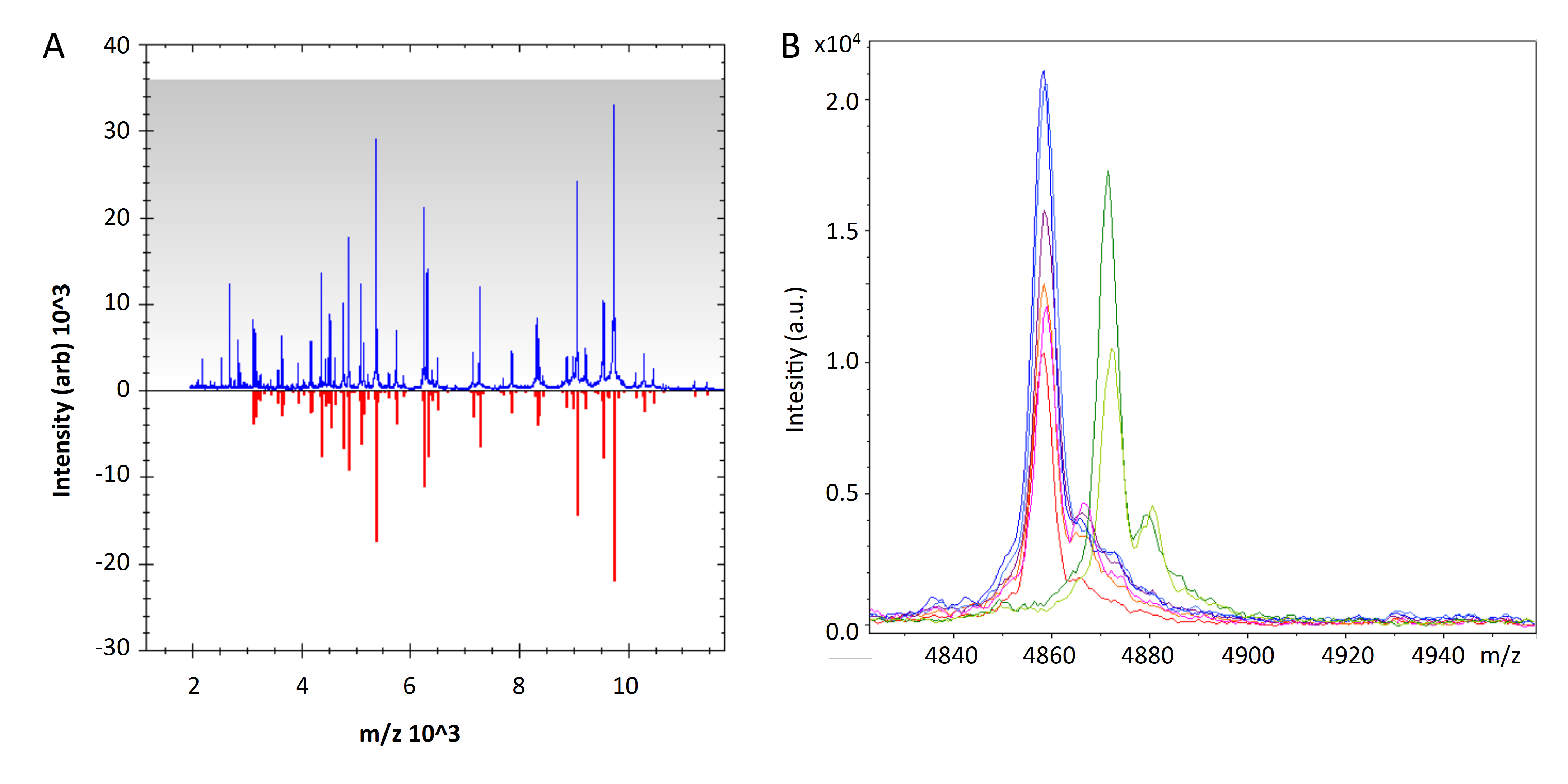

Supplement: S1 Fig — Blue lines show MS peaks of an individual isolate. Red lines indicate the reference peaks. B. Representative example of mass spectrum differences between ESBL E. coli. Isolates are indicated by color code. Isolates 1, 5, 9, 13, 17 and 25 (outbreak cluster) show a clear distinguished peak at position 4859 m/z, whereas isolates 21 and 29 show a shift to position 4872 m/z (non-related cluster). This corresponds to change of about 13Da. (TIF) [file pone.0120624.s001.tif]

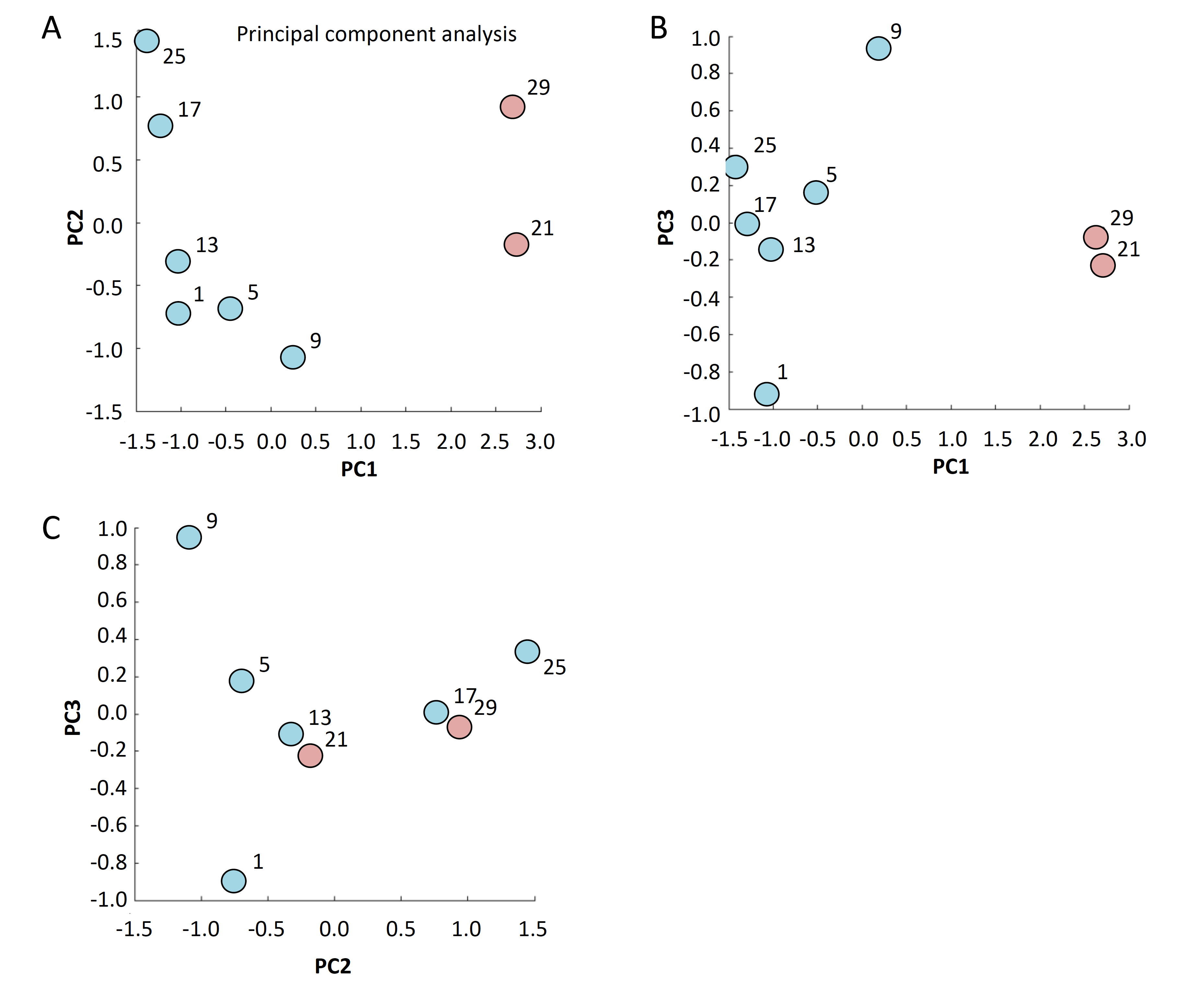

Supplement: S2 Fig — The influence on the data distribution by PC1, PC2 and PC3 is 70%, 20%, and 10% respectively. (TIF) [file pone.0120624.s002.tif]
