# Supplementary material for: Matrix-Assisted Laser Desorption/Ionization Time of Flight Mass-Spectrometry (MALDI-TOF MS) Based Typing of Extended-Spectrum β-Lactamase Producing E. coli – A Novel Tool for Real-Time Outbreak Investigation
Source: PLoS One. 2015 Apr 10;10(4):e0120624. doi: 10.1371/journal.pone.0120624 (PMC4393243; doi:10.1371/journal.pone.0120624)
Supplement: S1 Text — (DOCX) [file pone.0120624.s003.docx]

**Supplementary information: Standard operating procedure for MALDI-TOF based typing using a BRUKER MALDI-TOF system.**

A detailed analysis of mass spectra allows the identification of differences on a subspecies levels, which can be used for typing.

**A. Required reagents**

| Name |  |
| --- | --- |
| Ethanol, absolute >99.8% |  |
| Formic acid, 70% | 300uL ultrapure water and 700uL formic acid (puris, p.a. 98%) |
| Acetonitril, LC-MS CHROMASOLV >99.9% |  |
| Matrix solution | Standard solution is added to lyophilized a-cyano-4-hydroxycinnamic acid (HCCA) |
| Water, CHROMASOLV HPLC |  |
| Standard solution | 50% acetonitrile, 47.5% water, 2.5% trifluoroacetic acid (TFA) |

**B. Collection of bacterial isolates**

All tested isolates should be sub-cultured at the same time on standard blood agar plates. Following overnight cultivation (16h to 20h), a small aliquot of bacteria (2-3 colonies) is collected for each isolate separately. All isolates have to be collected at the same time. Cultures older than 24h should not be used for typing analysis. Frozen isolates should be sub-cultured twice.

**C. Preparation and extraction**

1. Using a 1uL loop, 2-3 colonies should be picked and transferred in a tube with 300uL dH_2_0.

2. Vortex for 10 seconds.

3. Add 900uL of pure ethanol (absolute)

4. Short vortex

5. Centrifuge for 2 minutes at 13000U/min for pelleting.

6. Carefully discard supernatant, without losing the pellet. Centrifuge again for 2 min at 13000U/min.

7. Remove the remaining ethanol by carefully pipetting.

8. Let the pellet dry for 5 minutes (not longer than 10min).

9. Dependent on the pellet size add 15-20uL formic acid (70%). Dissolve the pellet by carefully pipetting up and down. Incubate the dissolved bacteria for 2 min.

10. Add the same volume (15-20uL) of acetonitrile (ratio 1:1) and mix.

11. Centrifuge for 2 min at 13000U/min

12. Add 1uL of the supernatant to spot on the MALDI target plate.

13. Let the spot dry (5-10min) and add 1uL Matrix solution.

14. Record four MALDI spectra in the FLEX control software for each isolate.

15. Species should be verified in the MALDI Biotyper 3 software.

16. Detailed peak analysis is performed in Flex Analysis 3.

17. List peaks with a Signal to noise ratio >10 and a minimum of 1000 a.u. Compare isolates against each other to determine outbreak – specific peaks

In the initial evaluation phase it is recommended to repeat the same isolates 3 times and see if isolate specific peaks can be reproduced.
