# Supplementary material for: Matrix-Assisted Laser Desorption/Ionization Time of Flight Mass-Spectrometry (MALDI-TOF MS) Based Typing of Extended-Spectrum β-Lactamase Producing E. coli – A Novel Tool for Real-Time Outbreak Investigation
Source: PLoS One. 2015 Apr 10;10(4):e0120624. doi: 10.1371/journal.pone.0120624 (PMC4393243; doi:10.1371/journal.pone.0120624)
Supplement: S1 Table — (DOCX) [file pone.0120624.s004.docx]

**Supplementary Table 1.** List of all identified peaks. All mass spectra of ESBL *E.coli* with a signal-to-noise ratio >10 and a minimum peak intensity of 1000 a.u. are shown. Double charged peaks are indicated (*).

|  | **Mass spectra peaks (m/z)** | | | | | | | | | | |
| --- | --- | --- | --- | --- | --- | --- | --- | --- | --- | --- | --- |
| **Isolates** | 4165* | 4175* | 4859* | 4872* | 6274 | 8328 | 8353 | 9716 | 9743 | 10467 | 10494 |
| 1 |  | **X** | **X** |  |  |  | **X** | **X** |  |  | **X** |
| 5 |  | **X** | **X** |  |  |  | **X** | **X** |  |  | **X** |
| 9 |  | **X** | **X** |  |  |  | **X** | **X** |  |  | **X** |
| 13 |  | **X** | **X** |  |  |  | **X** | **X** |  |  | **X** |
| 17 |  | **X** | **X** |  |  |  | **X** | **X** |  |  | **X** |
| 25 |  | **X** | **X** |  |  |  | **X** | **X** |  |  | **X** |
| 21 | **X** |  |  | **X** | **X** | **X** |  |  | **X** | **X** |  |
| 29 | **X** |  |  | **X** | **X** | **X** |  |  | **X** | **X** |  |

Outbreak isolates: 1, 5, 9, 13, 17, and 25; non-related isolated 21-29.
